# Orthodenticle Homeobox OTX1 Promotes Papillary Thyroid Carcinoma Progression and Is a Potential Prognostic Biomarker

**DOI:** 10.1155/2023/5513812

**Published:** 2023-09-21

**Authors:** Jing Wei, Xin Wang, Kai Jiao

**Affiliations:** ^1^Department of Endocrinology, Xi'an Gaoxin Hospital, Xi'an 710077, China; ^2^Department of Endocrinology, Tangdu Hospital, Xi'an 710038, China

## Abstract

Papillary thyroid carcinoma (PTC) is the most common type of thyroid neoplasms, characterized by evidence of follicular cell differentiation. Orthodenticle homeobox 1 (OTX1) is a transcription factor which has been implicated in numerous diseases, including malignancies. The objective of this research was to explore the function of OTX1 in PTC. Immunohistochemistry (IHC) was employed to determine the protein level of OTX1 in PTC specimens. Cell viability was assessed by the 3-(4,5-dimethylthiazol-2-yl)-2,5-diphenyltetrazolium bromide (MTT) assay. Furthermore, a xenograft model on nude mice was established to investigate in vivo effects of OTX1. Our results revealed that OTX1 was significantly upregulated within specific PTC tissues and was remarkably correlated with unfavorable clinical outcomes in PTC. Silencing OTX1 resulted in a significant inhibition in cell viability and suppressed cell proliferation. In addition, in vivo experiments demonstrated that OTX1 silencing resulted in a significant suppression of tumor growth in nude mice. Collectively, these results suggest that OTX1 may play crucial roles in promoting PTC progression.

## 1. Introduction

Papillary thyroid carcinoma (PTC) represents the most prevalent type of thyroid cancers, accounting for about 80% of all thyroid malignancies [1]. It is characterized by distinctive nuclear features, including nuclear pseudoinclusions and nuclear grooves [2]. Despite being generally associated with a favorable prognosis, a subset of PTC cases exhibits aggressive behavior and increased risk of recurrence [3]. Therefore, there is a critical need to identify molecular markers that can aid in predicting the prognosis and guiding treatment decisions for PTC patients.

Transcription factors are crucial regulators of gene expression that play pivotal roles in almost all cellular processes, including cellular proliferation, differentiation, and survival [4]. One such transcription factor is orthodenticle homeobox 1 (OTX1), which belongs to the homeobox gene family [5]. Homeobox genes encode transcription factors categorized by a conserved DNA-binding domain known as the homeodomain, enabling them to bind to specific DNA sequences and regulate expression of target genes [6]. OTX1 is involved in important developmental processes, including embryonic development, neurogenesis, and organogenesis [7]. It serves as a transcriptional regulator, influencing the expression of genes necessary for proper tissue and organ formation during embryogenesis [8]. However, aberrant expression of OTX1 has been observed in various cancers, such as lung cancer and colorectal cancer [9, 10]. In bladder cancer, OTX1 dysregulation has been linked to increased tumor cell proliferation and invasiveness [11]. Similarly, in lung cancer, abnormal OTX1 expression has been associated with enhanced invasion capability of tumor cells, contributing to metastasis [12]. In addition, an aberrant OTX1 level has been indicated in cervical cancer progression [13].

Despite these insights into OTX1's involvement in several cancers, its specific role in PTC remains inadequately understood. PTC is the most common type of thyroid cancer characterized by differentiated follicular cells. Given the importance of transcription factors in cellular processes and the emerging role of OTX1 in other cancers, investigating its role in PTC could provide valuable insights into the underlying molecular mechanisms driving PTC development and progression.

Therefore, the objective of the current study was to explore the expression pattern of OTX1 in PTC tissues and assess its correlations with clinicopathological characteristics. In addition, we aimed to examine the functional significance of OTX1 in PTC by investigating its impact on cell viability and growth. By conducting these investigations, we aimed to gain a deeper understanding of the roles of OTX1 in PTC and explore its clinical significance as a prognostic biomarker as well as a potential therapeutic target, specifically in the context of PTC.

## 2. Methods

### 2.1. Patient Samples and Immunohistochemistry (IHC) Detection

A cohort of PTC patient samples (*n* = 294) was collected from Xi'an Gaoxin Hospital. The inclusion criteria for the study encompassed histologically confirmed PTC cases in patients aged between 18 and 80 years, exclusively PTC cases without distant metastasis, and individuals who underwent surgical resection. Data were collected from PTC patients diagnosed within the period of 2010–2015, and only patients with a recorded survival month greater than zero were considered. IHC was performed to evaluate the expression of OTX1 in PTC tissues as previously reported using the antibody from Abcam (#ab25985; 1 : 200 dilution) [14].

### 2.2. Cell Culture and Transfection

Human PTC cell lines (BCPAP and TPC1) were cultured under standard conditions. Cells were infected with OTX1-shRNA or negative control scrambled shRNA using lentiviral shRNAs [15]. The following shRNA sequences were used: shOTX1#1: GCAACACCTCGTGTATGCA; shOTX1#2: GCCGACTGCTTGGATTACA [16].

### 2.3. Cell Viability Assay

Cell viability was assessed using the 3-(4,5-dimethylthiazol-2-yl)-2,5-diphenyltetrazolium bromide (MTT) assay according to the manufacture's procedure. In total, 5,000 cells per well per 200 *μ*L were seeded to start the cell proliferation test.

### 2.4. Xenograft Model

Nude mice were subcutaneously injected with PTC cells infected with OTX1-shRNA or negative control shRNA. Tumor growth was monitored every five days, and tumor volume was calculated. After one month, xenografts were isolated for weighing. In total, 500,000 cells per 100 *μ*L were seeded subcutaneously to start the xenograft experiment.

### 2.5. Statistical Analysis

Descriptive statistics were used to describe patient characteristics such as age, sex, laterality, tumor diameter, differentiation grade, T stage, N stage, surgery type, radiotherapy status, and OTX1 expression level. To assess the relationship between these variables and survival outcomes, Kaplan–Meier survival analyses were conducted, providing information on mean survival months, 5-year cancer-specific survival (CSS) percentages, and corresponding *p* values. The significance of the associations was evaluated using the chi-square test or Fisher's exact test for categorical variables and the *t*-test for cellular and animal experiments compared to the “scrambled-shRNA” group [17]. SPSS and Prism software packages were employed in this study.

### 2.6. Ethics Approval

This study was conducted at Xi'an Gaoxin Hospital in compliance with the ethical guidelines and regulations set forth by the Institutional Review Board (IRB). Prior ethical approval was obtained to ensure the protection of human subjects and animal welfare. Informed consent was obtained from all participants, emphasizing voluntary participation, understanding of the study objectives and procedures, and the right to privacy and confidentiality. Patient ethics were upheld by ensuring anonymity, confidentiality, and the responsible use of collected data solely for research purposes. Animal ethics were strictly followed, adhering to the guidelines of the IACUC, ensuring appropriate housing, care, and handling. By obtaining ethical approval, obtaining informed consent, and prioritizing animal welfare, this study demonstrated a commitment to upholding ethical standards in research while safeguarding the rights and well-being of human participants and animal subjects.

## 3. Results

### 3.1. Patients' Information

Table 1 displays the comprehensive information of the 294 patients diagnosed with papillary thyroid carcinoma (PTC) included in this study. The patients' characteristics are presented in terms of age, sex, laterality, tumor diameter, differentiation grade, T stage, N stage, surgery type, and radiotherapy. The cohort consisted of an equal distribution between patients younger than 49 years (50.0%, *n* = 147) and those aged 49 years or older (50.0%, *n* = 147). The majority of patients were female (74.8%, *n* = 220) compared to males (25.2%, *n* = 74). Tumor distribution indicated 43.2% (*n* = 127) in the left lobe, 48.6% (*n* = 143) in the right lobe, and 8.2% (*n* = 24) in bilateral lobes. Tumor diameter was divided into <1.7 cm (50.0%, *n* = 147) and ≥1.7 cm (50.0%, *n* = 147). Differentiation grade revealed that PTC cases were predominantly well differentiated (78.9%, *n* = 232), with fewer cases classified as moderately differentiated (12.9%, *n* = 38) or poorly differentiated (8.2%, *n* = 24). T-stage distribution showed T1 (50.7%, *n* = 149), T2 (18.7%, *n* = 55), T3 (24.8%, *n* = 73), and T4 (5.8%, *n* = 17). In terms of lymph node involvement (N stage), the majority were classified as N0 (81.0%, *n* = 238), with smaller proportions of N1a (11.2%, *n* = 33) and N1b (7.8%, *n* = 23). Surgery types included lobectomy and/or isthmectomy (16.7%, *n* = 49), subtotal or near total thyroidectomy (3.4%, *n* = 10), and total thyroidectomy (79.9%, *n* = 235). Regarding radiotherapy, 57.5% (*n* = 169) of patients did not receive radiotherapy, while 42.5% (*n* = 125) accepted postoperative radiotherapy as part of their treatment.

### 3.2. Expression of OTX1 in PTC Tissues

**Figure 1:**
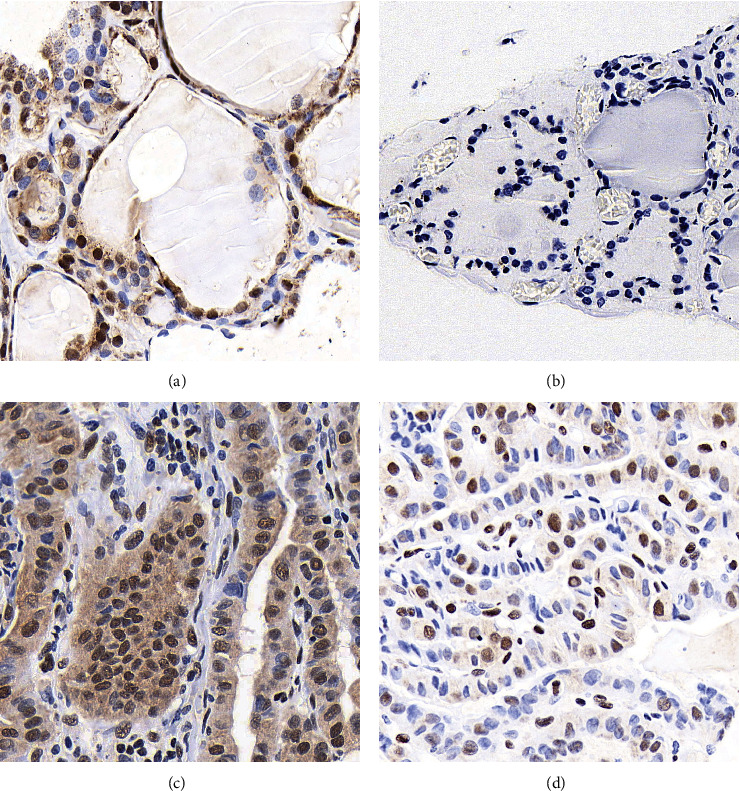
Representative IHC staining results. Representative IHC staining images were present for high OTX1 in adjacent thyroid tissue (a), negative OTX1 in adjacent thyroid tissue (b), high OTX1 in PTC tissue (c), and low OTX1 in PTC tissue (d) (magnification: 400x).

Immunohistochemical analysis revealed a high expression of OTX1 in 46.9% of PTC tissues (*n* = 138), whereas 81.0% of adjacent nontumor tissues (*n* = 238) exhibited weak or negative staining for OTX1 (Figure 1).  Table 2 provides a comprehensive analysis of the correlation between OTX1 expression levels and various characteristics of the 294 PTC patients included in this study. No significant correlations were found between OTX1 expression and age or sex. Similarly, no significant association was detected between OTX1 levels and laterality or surgery type. However, a significant correlation was identified between OTX1 expression and tumor diameter, with a higher expression in PTC patients with smaller tumor sizes (<1.7 cm, *p*=0.010). Furthermore, OTX1 expression was marginally associated with the T stage, indicating a higher expression in advanced T stages (*p*=0.051). Notably, a significant correlation was found between OTX1 expression and lymph node involvement (N stage), with a higher expression observed in patients with lymph node metastasis (*p*=0.007). No significant correlations were observed between OTX1 expression and differentiation grade or radiotherapy status. These findings indicated a critical role of OTX1 in PTC progression, particularly in tumor size determination and lymph node metastasis. Nevertheless, more investigations are essential to unravel underlying mechanisms and clinical implications of these associations.

### 3.3. OTX1 Indicates Poor PTC Survival

**Figure 2:**
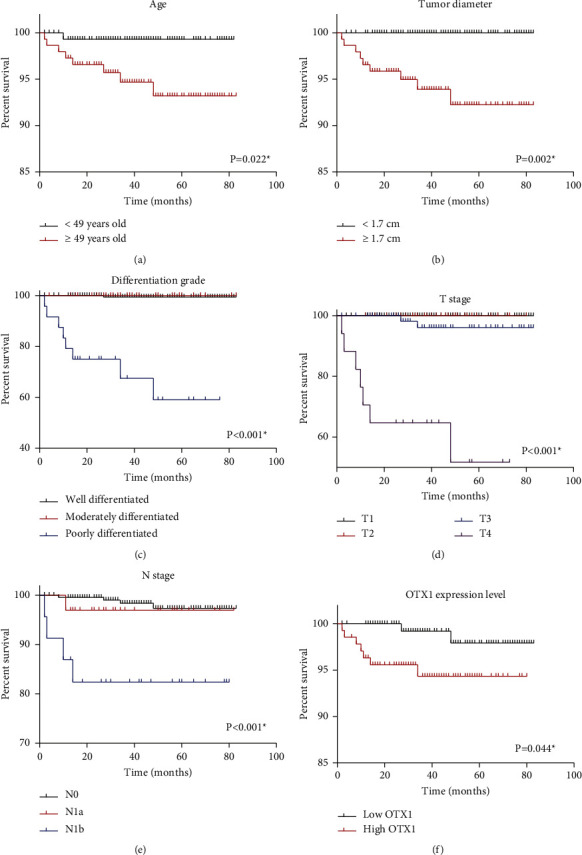
Cancer-specific survival of the enrolled PTC cohort. Kaplan–Meier survival analyses and log-rank tests were conducted for the enrolled PTC cohort based on patients' age (a), tumor diameter (b), differentiation grade (c), T stage (d), N stage (e), and OTX1 protein level (f).

Table 3 presents the results of Kaplan–Meier survival analyses conducted on a cohort of 294 patients with PTC. The analysis revealed several significant associations between these variables and survival outcomes. Notably, younger patients (<49 years) had a significantly higher mean survival time (81.5 ± 0.5 months) and 5-year cancer-specific survival (CSS) rate (99.3%) than older patients (≥49 years) (78.9 ± 1.4 months, 93.2% CSS, Figure 2(a)). In addition, tumor diameter was found to be significantly correlated with survival, with patients having tumors smaller than 1.7 cm showing a 100% 5-year CSS than those with larger tumors (92.3% CSS, Figure 2(b)). Other significant associations were observed between survival outcomes and the differentiation grade, T stage, N stage, and OTX1 expression level. Well-differentiated tumors had the highest 5-year CSS (99.5%), while poorly differentiated tumors had the lowest (59.1%, Figure 2(c)). Similarly, patients with lower T (Figure 2(d)) and N stages (Figure 2(e)) exhibited better survival rates. Interestingly, a low OTX1 expression was associated with a higher 5-year CSS (97.9%) compared to high OTX1 expression (94.3%, Figure 2(f)). The reason for missing survival times in certain subgroups (Table 3) is due to the 100% 5-year survival rate observed in these particular groups. As a result, the median survival time cannot be calculated for these specific groups. The 100% 5-year survival rate indicates that all patients in these groups survived for at least 5 years from the time of diagnosis, making the calculation of the median survival time impractical. Nevertheless, these findings highlight the prognostic significance of age, tumor characteristics, and OTX1 expression in PTC, providing valuable insights for risk stratification and treatment decision-making in clinical practice.

### 3.4. OTX1 Silencing Inhibits Cell Viability and Growth

**Figure 3:**
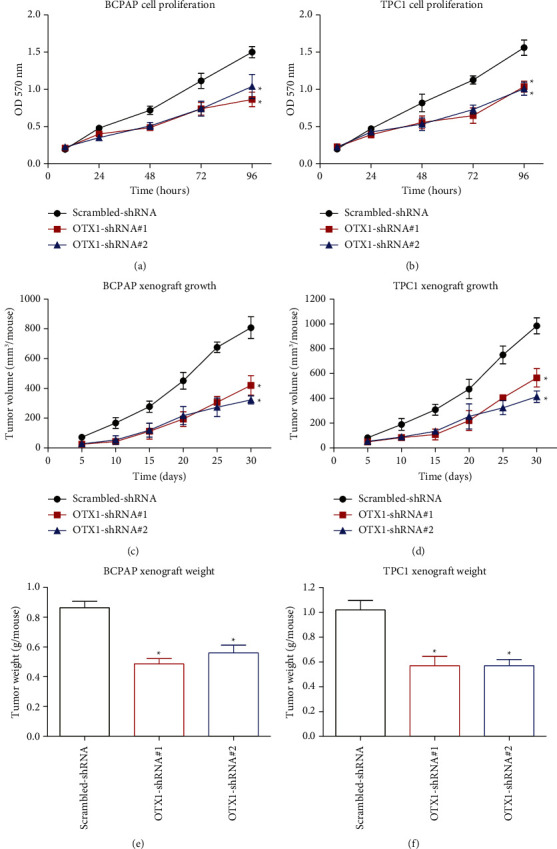
Effects of OTX1 silencing on cell growth and tumor progression in papillary thyroid carcinoma (PTC). (a, b) Cell viability assays revealed a significant reduction in cell growth following OTX1 silencing in both BCPAP and TPC1 PTC cell lines. (c, d) In vivo xenograft experiments using nude mice demonstrated slower subcutaneous tumor growth upon OTX1 silencing, with tumors generated from BCPAP and TPC1 cell lines, respectively. (e, f) Analysis of excised xenografts showed a notable decrease in tumor weight in the OTX1-shRNA group compared to that in the scrambled-shRNA group. These findings provide compelling evidence for the crucial role of OTX1 in promoting cell growth and tumor progression in PTC, supporting its potential as a therapeutic target. The error bars represent the standard deviation. Student's *t*-test was conducted, and statistical significance was denoted as  ^*∗*^*p* < 0.05.

We further provided compelling evidence regarding the impact of OTX1 on cell growth in PTC. MTT results clearly demonstrate a significant reduction in cell growth upon silencing OTX1 in both the BCPAP and TPC cell lines (Figures 3(a) and 3(b)). These observations highlight the crucial role of OTX1 in promoting the proliferation of PTC cells.

To corroborate the in vitro results, xenograft experiments were performed using nude mice to assess the effect of OTX1 silencing on tumor growth in a physiological setting. Strikingly, the xenografts derived from the OTX1-silenced BCPAP and TPC cell lines exhibited significantly slower growth rates than those from the control group (Figures 3(c) and 3(d)). Consistently, upon excising the xenografts, the OTX1-shRNA group exhibited a substantial reduction in tumor weight compared to the scrambled-shRNA group (Figures 3(e) and 3(f)). These findings provide further support for the inhibitory effect of OTX1 silencing on tumor growth in an in vivo context.

In summary, the combined results from the in vitro and in vivo experiments consistently indicate a pivotal role of OTX1 in promoting PTC tumor growth and tumor progression. These results unveiled the significance of OTX1 as a promising treatment target for interventions aimed at restraining the growth and progression of papillary thyroid carcinoma. Further investigations elucidating the underlying molecular mechanisms through which OTX1 influences PTC development and progression are warranted to enhance our understanding of its functional significance in this malignancy.

## 4. Discussion

PTC is the most common type of thyroid neoplasm, characterized by its association with follicular cell differentiation. Here, we aimed to explore the significance of orthodenticle homeobox 1 (OTX1), a member of the homeobox gene family and a transcription factor, in PTC progression and its potential as a prognostic biomarker. Our findings contribute to the understanding of OTX1's involvement in PTC and align with the existing literature on the functional significance of transcription factors in cancer [18–20].

Transcription factors play pivotal roles in regulating gene expression and orchestrating various biological processes, including cellular proliferation, differentiation, and survival. OTX1, a transcription factor, has been implicated in embryonic development, neurogenesis, and organogenesis [21]. Moreover, its dysregulated expression has been discovered in several cancer types including ovarian cancer, prostate cancer, and colorectal cancer [9, 22, 23]. These studies highlight the versatile role of OTX1 in different disease contexts, including cancer.

Immunohistochemistry (IHC) analysis of PTC tissues revealed a significantly higher OTX1 expression in certain tumor samples, indicating its potential as a prognostic biomarker in PTC. This finding is consistent with previous data demonstrating the clinical relevance of OTX1 expression in cancer. For instance, research conducted on hepatocellular carcinoma has shown that an elevated OTX1 expression is associated with a poor prognosis and tumor progression [24]. In addition, in lung cancer, OTX1 overexpression has been linked to advanced disease stage and reduced survival [10]. Collectively, these findings underscore the significance of OTX1 as a potential prognostic indicator in various cancer types.

In vitro experiments using PTC cell lines further elucidated the functional role of OTX1 in PTC progression. OTX1 silencing resulted in reduced cell viability and inhibited cell growth, suggesting its crucial involvement in promoting PTC cell proliferation. These findings align with those of previous studies on OTX1 in cancer. In lung cancer, OTX1 knockdown has been shown to suppress cell growth and induce apoptosis, highlighting its potential as a therapeutic target [10]. Similarly, in laryngeal squamous cell carcinoma, targeting OTX1 impairs tumor cell proliferation and tumor growth [16]. These studies provide additional support for the functional significance of OTX1 in cancer cell biology.

In vivo xenograft experiments using nude mice corroborated the in vitro findings, demonstrating that silencing OTX1 significantly suppressed tumor growth in subcutaneous PTC xenografts derived from BCPAP and TPC cell lines. This observation is in line with that of previous studies implicating OTX1 in tumor progression. In esophageal squamous cell carcinoma, overexpressing OTX1 has been shown to promote tumor growth as well as invasion in nude mice [25]. Moreover, in pancreatic cancer, targeting OTX1 has been associated with reduced tumor growth and metastasis [26]. These studies underscore the importance of targeting OTX1 as a therapeutic direction to inhibit tumor progression in various cancer types.

In addition, the Kaplan–Meier survival analyses demonstrated a significant association between high OTX1 expression and reduced cancer-specific survival in PTC cases. This finding is consistent with previous results indicating the clinical relevance of OTX1 in cancer prognosis. In gastric cancer, an elevated OTX1 expression has been correlated with worse survival [19]. Similarly, in ovarian cancer, a high OTX1 level has been associated with advanced disease stage and unfavorable prognosis [23]. These studies collectively emphasize the significance of OTX1 as a prognostic marker across different cancer types.

This study significantly advances our understanding of OTX1's role in thyroid cancer, particularly papillary thyroid carcinoma (PTC). First, this study demonstrates that OTX1 is significantly upregulated in PTC tissues. This is a significant finding because it suggests that OTX1 plays a role in the development or progression of PTC, offering a new avenue for understanding the molecular mechanisms of this type of cancer. Second, we also show that a high OTX1 expression is correlated with unfavorable clinical outcomes in PTC patients. This suggests that OTX1 could potentially be used as a prognostic biomarker for PTC, helping physicians predict disease progression and potentially tailor treatment strategies accordingly. Moreover, our data show that silencing OTX1 leads to a significant decrease in cell viability and proliferation and suppresses tumor growth in a mouse model, therefore providing valuable insights into the functional role of OTX1 in PTC and suggesting that OTX1 could be a potential therapeutic target in PTC treatment. Given the correlation between OTX1 expression and clinical outcomes, OTX1 could be developed as a prognostic biomarker for PTC. Developing drugs or therapies that can inhibit or modulate OTX1 activity could potentially be an effective strategy for treating PTC. In addition, understanding the molecular pathways and mechanisms through which OTX1 acts could lead to the discovery of other potential therapeutic targets or strategies.

## 5. Conclusion

Our study provides evidence for the oncogenic role of OTX1 in papillary thyroid carcinoma (PTC). The high expression of OTX1 in PTC tissues is significantly associated with a worse prognosis. Moreover, OTX1 silencing impairs cell viability and growth both in vitro and in vivo. Our findings suggest that OTX1 may serve as a potential prognostic biomarker and therapeutic target for PTC. Further studies are needed to elucidate the underlying molecular mechanisms and explore the clinical implications of targeting OTX1 in PTC management.

## Figures and Tables

**Table 1:** PTC patients' information.

Variables	Cases (*n* = 294)	Frequencies
*Age (years)*		
<49	147	50.0
≥49	147	50.0
*Sex*		
Female	220	74.8
Male	74	25.2
*Laterality*		
Left lobe	127	43.2
Right lobe	143	48.6
Bilateral	24	8.2
*Tumor diameter (cm)*		
<1.7	147	50.0
≥1.7	147	50.0
*Differentiation grade*		
Well differentiated	232	78.9
Moderately differentiated	38	12.9
Poorly differentiated	24	8.2
*T stage*		
T1	149	50.7
T2	55	18.7
T3	73	24.8
T4	17	5.8
*N stage*		
N0	238	81.0
N1a	33	11.2
N1b	23	7.8
*Surgery*		
Lobectomy and/or isthmectomy	49	16.7
Subtotal or near total thyroidectomy	10	3.4
Total thyroidectomy	235	79.9
*Radiotherapy*		
Absent	169	57.5
Accepted	125	42.5

**Table 2:** Correlations between OTX1 expression and PTC patients' characteristics.

Variables	Cases (*n* = 294)	OTX1 level		*p* value
		Low (*n* = 156)	High (*n* = 138)	
*Age (years)*				
<49	147	74	73	0.350
≥49	147	82	65	
*Sex*				
Female	220	116	104	0.843
Male	74	40	34	
*Laterality*				
Left lobe	127	69	58	0.746
Right lobe	143	76	67	
Bilateral	24	11	13	
*Tumor diameter (cm)*				
<1.7	147	89	58	0.010^*∗*^
≥1.7	147	67	80	
*Differentiation grade*				
Well differentiated	232	129	103	0.173
Moderately differentiated	38	15	23	
Poorly differentiated	24	12	12	
*T stage*				
T1	149	90	59	0.051
T2	55	24	31	
T3	73	36	37	
T4	17	6	11	
*N stage*				
N0	238	136	102	0.007^*∗*^
N1a	33	14	19	
N1b	23	6	17	
*Surgery*				
Lobectomy and/or isthmectomy	49	27	22	0.850
Subtotal or near total thyroidectomy	10	6	4	
Total thyroidectomy	235	123	112	
*Radiotherapy*				
Absent	169	94	75	0.306
Accepted	125	62	63	

^*∗*^*p* < 0.05.

**Table 3:** Kaplan–Meier survival analyses of the enrolled PTC cohort.

Variables	Cases (*n* = 294)	Survival months (mean ± S.D.)	5-year CSS (%)	*p* value
*Age (years)*				
<49	147	81.5 ± 0.5	99.3	0.022^*∗*^
≥49	147	78.9 ± 1.4	93.2	
*Sex*				
Female	220	81.3 ± 0.8	97.6	0.190
Male	74	78.9 ± 2.0	92.2	
*Laterality*				
Left lobe	127	81.2 ± 1.0	97.4	0.828
Right lobe	143	80.4 ± 1.2	95.1	
Bilateral	24	73.9 ± 3.1	95.8	
*Tumor diameter (cm)*				
<1.7	147	—	100	0.002^*∗*^
≥1.7	147	—	92.3	
*Differentiation grade*				
Well differentiated	232	—	99.5	<0.001^*∗*^
Moderately differentiated	38	—	100	
Poorly differentiated	24	—	59.1	
*T stage*				
T1	149	—	100	<0.001^*∗*^
T2	55	—	100	
T3	73	—	96.1	
T4	17	—	51.8	
*N stage*				
N0	238	81.7 ± 0.6	97.3	<0.001^*∗*^
N1a	33	79.8 ± 2.1	97.0	
N1b	23	67.2 ± 5.8	82.4	
*Surgery*				
Lobectomy and/or isthmectomy	49	—	97.9	0.708
Subtotal or near total thyroidectomy	10	—	100	
Total thyroidectomy	235	—	95.5	
*Radiotherapy*				
Absent	169	81.1 ± 0.9	96.4	0.469
Accepted	125	80.3 ± 1.3	95.6	
*OTX1 expression level*				
Low	156	82.1 ± 0.6	97.9	0.044^*∗*^
High	138	76.2 ± 1.4	94.3	

^*∗*^*p* < 0.05.

## Data Availability

The data that support the findings of this study are available from the corresponding author upon reasonable request.
